# A new attribute-linked residential property price dataset for England and Wales, 2011–2019

**DOI:** 10.14324/111.444/ucloe.000019

**Published:** 2021-05-27

**Authors:** Bin Chi, Adam Dennett, Thomas Oléron-Evans, Robin Morphet

**Affiliations:** 1 Centre for Advanced Spatial Analysis (CASA), University College London, 90 Tottenham Court Road, London W1T 4TJ, UK

**Keywords:** Land Registry Price Paid Data, Domestic Energy Performance Certificates, data linkage, England and Wales

## Abstract

Current research on residential house price variation in the UK is limited by the lack of an open and comprehensive house price database that contains both transaction price alongside dwelling attributes such as size. This research outlines one approach which addresses this deficiency in England and Wales through combining transaction information from the official open Land Registry Price Paid Data (LR-PPD) and property size information from the official open Domestic Energy Performance Certificates (EPCs). A four-stage data linkage is created to generate a new linked dataset, representing 79% of the full market sales in the LR-PPD. This new linked dataset offers greater flexibility for the exploration of house price (£/m^2^) variation in England and Wales at different scales over postcode units between 2011 and 2019. Open access linkage codes will allow for future updates beyond 2019.

## Introduction

Comparative international analyses of house prices are constrained by differences in definition, data structure, spatial/time scales and coverage. These limit both comparative analysis and within-country analysis of housing markets [1,2]. House price data deficiencies hinder research on residential house price variation in the UK, and limit understanding of the housing market. Modelling of UK-based house price changes dates back to the 1970s [3,4] with much of the data used either aggregated to coarse geographies such as regions or districts or, conversely, associated with individual properties in a specific city. Aggregate sample mortgage data, mainly from building societies, such as the 5% sample survey of Building Society Mortgages and the Nationwide Building Society mortgage data, have been widely used [5–13]. These datasets lack local nuance but are also problematic due to the potential biases inherent in small samples [14,15]. Conversely, more detailed micro-level housing data such as the local estate agent survey data used by Orford [16] have offered opportunities for local housing analysis, but such datasets are not widely available.

Land Registry Price Paid Data (LR-PPD) have been published as open data since 2013. These data have been transformative for house price variation research in the UK [17–20] as they are a comprehensive record of residential transactions at address level in England and Wales dating back to 1995 [21]. Although the Land Registry excludes some types of residential property sales (e.g. ‘Right to buy’ sales at a discount), these data still provide the most accurate picture of residential property sales at full market value in England and Wales [22]. The Office for National Statistics (ONS) has used the LR-PPD to calculate official house price statistics such as the House Price Statistics for Small Areas dataset [23] and the official House Price Index [24]. Despite the utility of these data a lack of attribute information relating to the properties, such as total floor size information, is identified as one of the major shortcomings, as the impacts of stock mix on broader patterns cannot be fully accounted for [12,25].

As total floor area is identified as the most important determinant of house price variation [25–28], two approaches have been developed in the UK to enhance the LR-PPD by adding total floor area. The first approach, created by Orford [25], adds an estimated total floor area to the LR-PPD. The estimated total floor area is derived from building footprints obtained from Ordnance Survey MasterMap and Environment Agency LiDAR data, but the methods are recognised as problematic for estimating the floor area of flats within a building [25], and for properties where the number of stories cannot be accurately inferred.

The second approach is more direct and links LR-PPD with the total floor area information from Domestic Energy Performance Certificates [29–33]. Domestic Energy Performance Certificates (Domestic EPCs) is an open dataset released by the Ministry for Housing, Communities and Local Government (MHCLG). It not only records a property’s energy performance but also gives building attribute information (i.e., total floor area or number of habitable rooms). Despite this link being feasible, only two research studies have mentioned the linkage rate between LR-PPD and Domestic EPCs and no research has yet published the details of both the linkage method and linkage data [32,33]. Aiming to remedy this situation, we publish our own linkage codes alongside the open access and reusable house price per square metre dataset.

## Data description and development

### LR-PPD and Domestic EPCs data

The LR-PPD dataset is open, available online and updated on a monthly basis (https://www.gov.uk/government/statistical-data-sets/price-paid-data-downloads). The LR-PPD used in this research was downloaded in 2019 and contains 16 items with 24,852,949 transactions in England and Wales between 1/1/1995 and 31/10/2019. For each transaction, there is a unique transaction identifier along with the property’s transaction price, transaction date, address information (postcode, PAON, SAON, street), property type (detached, semi-detached, terraced houses or flats/maisonettes), whether a property is newly built or whether it was sold at full market value [21]. For various reasons, not all the properties within the dataset are sold at full market value, therefore these entries are excluded from the linkage exercise. These excluded entries comprise only 2.90% of the whole dataset.

EPCs have been required by law since 2008 for all properties sold, built or rented in England and Wales. Data from these certificates is open and available on-line from the MHCLG (https://epc.opendatacommunities.org/). The EPC dataset used in this research is the third version downloaded on 20/10/2020 and contains certificates issued between 1/10/2008 and 31/5/2019 [34]. It records 18,575,357 energy performance data records with 84 fields. It not only records a property’s energy performance but also building stock information, such as its address, total floor area and number of habitable rooms.

### Data linkage

**Figure 1:**
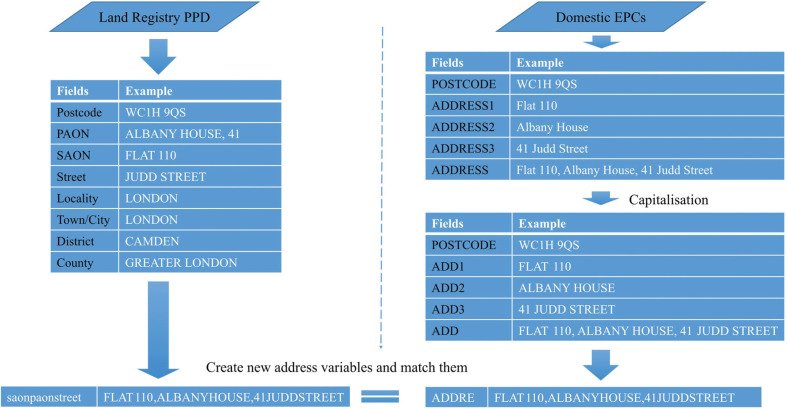
An example of the data linkage process.

The data linkage method used here is similar to an earlier published method [35], but with greater granularity in the matching rules. Linkage between the PPD and Domestic EPC dataset is achieved though several phases dealing with successively more complex address matching challenges. Before matching, transactions in the LR-PPD without postcodes in the Domestic EPCs dataset are excluded – this accounts for 0.55% of the data – leaving a total of 23,999,656 transactions for matching. Figure 1 shows an example of the data linkage process, with the basic idea of linkage between these two datasets being to match by full postal delivery address (i.e., postcode plus detailed address strings). These two datasets both contain property information at address level but their address structures differ, thus basic data standardisation is needed. First, all address strings in the Domestic EPCs are capitalised and stored in new variables. These newly created address variables are used to achieve an initial data linkage. To deal with more complex subsequent linkage passes, 183 new variables are created in the LR-PPD and 99 new variables are created in the Domestic EPCs (Appendix A).

**Figure 2:**
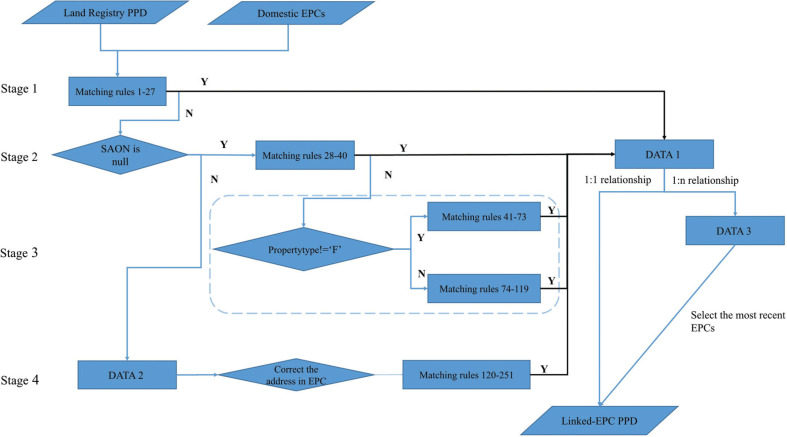
Workflow of the four-stage data linkage between LR-PPD and Domestic EPCs.

A matching method containing a four-stage (251 matching rules) process was designed and is outlined in Fig. 2. In the Domestic EPCs, each record is created using a unique identifier named id. Each transaction in the LR-PPD has a unique identifier named transactionid. Taking Stage 1 as an example of the matching process; all the matches are based on a temporary address string (i.e., postcode+saonpaonstreet) with the algorithm testing whether postcode+saonpaonstreet in LR-PPD is equal to any postcode +ADDRE in the Domestic EPCs. Where they match directly, records for both datasets are joined, removed from the original data and stored in a new temporary linked data table, DATA 1. For records where a match is not achieved on the first pass, the algorithm moves onto a further set of matching tests in Stage 2.

Problems emerge where one property may have more than one Domestic EPC. Where this is the case, only property transactions with just one successfully linked EPC will be moved from the temporary DATA 1 and directly stored in the final linked-EPC PPD dataset. Property transactions with successful links to more than one EPC are stored in a separate dataset, DATA 3. These data are filtered to select all Domestic EPCs for which total floor area is neither NULL nor 0 and then linked where the EPC inspection date or lodgement date is closest to the transaction date in the LR-PPD. This result will then be stored in the final linked-EPC PPD dataset. Stages 2 to 4 follow a similar process to Stage 1. The linked-EPC PPD dataset is the data linkage result. These data linkage results link back to the original Domestic EPCs and to the LR-PPD by their unique identifiers.

**Figure 3:**
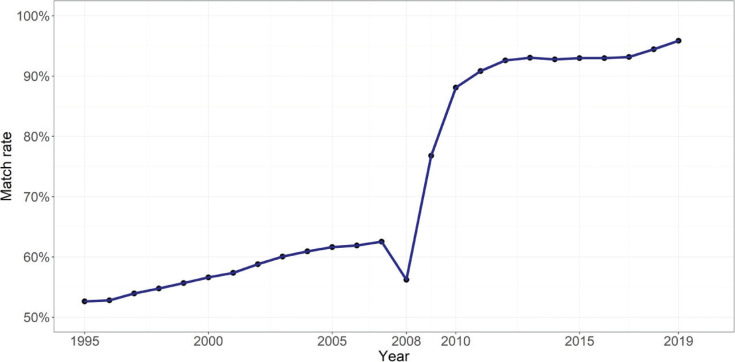
Match rate of linked house price data in England and Wales, 1995–2019.

Following the four-stage data linkage, 16,846,834 transaction records in England and Wales between 1995 and 2019 were successfully linked with Domestic EPCs. These comprise the linked dataset. The match rate of transactions in England is shown in Fig. 3. The match rate between 2011 and 2019 is higher than 90%, while the match rate of the rest of the period is considerably lower, this is mainly due to the EPCs dataset only covering the period between 1/10/2008 and 31/8/2019. The match rate of 56.20% in 2008 is particularly low but rapidly increases to over 88% after 2010. As the match rate before 2008 is significantly lower than for the period after 2008, only the linked data between 2009 and 2019 are used to conduct the evaluation of data linkage.

### Technical validation

#### Evaluation of the data linkage between 2009 and 2019

**Figure 4:**
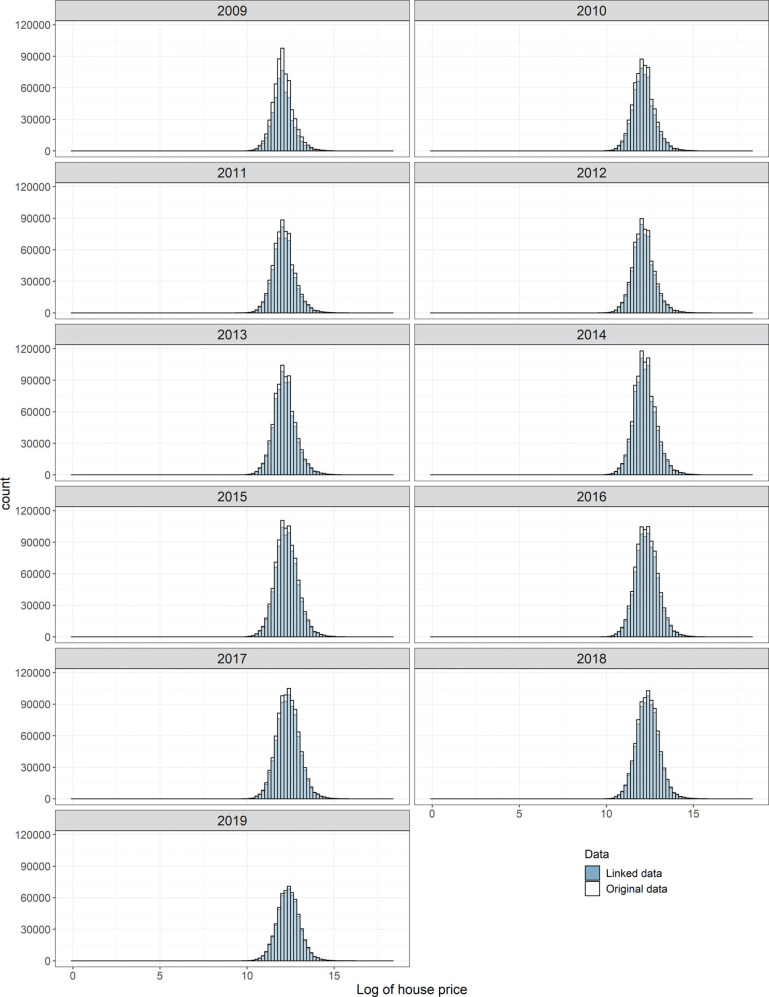
House price distribution of original data and linked data, 2009–2019.

Match rates offer a crude way to quantify the matching performance, but visual comparison of the house price frequency distributions for the new linked data and original LR-PPD data reveals a clearer picture of matching performance. Histograms of the logarithm of transaction price from both datasets are shown in Fig. 4. In each graph, the distribution of the linked data (blue) is overlaid onto the distribution of the original LR-PPD dataset (white). The area of visible white bars represents the proportion of un-matched cases. Importantly, there was no significant loss of information as a result of un-matched cases in the data linkage between 2010 and 2019.

The Kolmogorov–Smirnov test (K-S test) and the Jeffreys divergence (J-divergence) can be used to quantify the extent of house price information lost. The K-S test is a nonparametric test that examines the differences in the shape of a distribution. The K-S test, statistic D, is based on the maximum absolute difference between two cumulative distribution functions. Here, the test will be used to quantify the difference of two house price distributions (original data vs. linked data). The Jeffreys divergence (J-divergence), derived from information theory, is a function used to establish the distance of one probability distribution to another [36–38]. To calculate the J-divergence, the data from two different samples must first be assigned to *k* different categories. In the case of this research, these categories are a simple subdivision of the log house price into bins. The J-divergence is then defined as

(1) $$J=\sum_{j=1}^{k}p^{j}\ln\left(\frac{p^{j}}{q^{j}}\right)+\sum_{j=1}^{k}q^{j}\ln\left(\frac{q^{j}}{p^{j}}\right)$$

where *k* is the number of categories, *p^j^* is the proportion of data points in category *j* in the original house price data, and *q^j^* is the proportion of data points in category *j* in the linked house price data. The final divergence measure, *J*, ranges from 0 to 1. If the distribution of both data samples across all the categories is the same, *J* will be 0. Larger values of *J* indicate greater differences between the two distributions.

**Figure 5:**
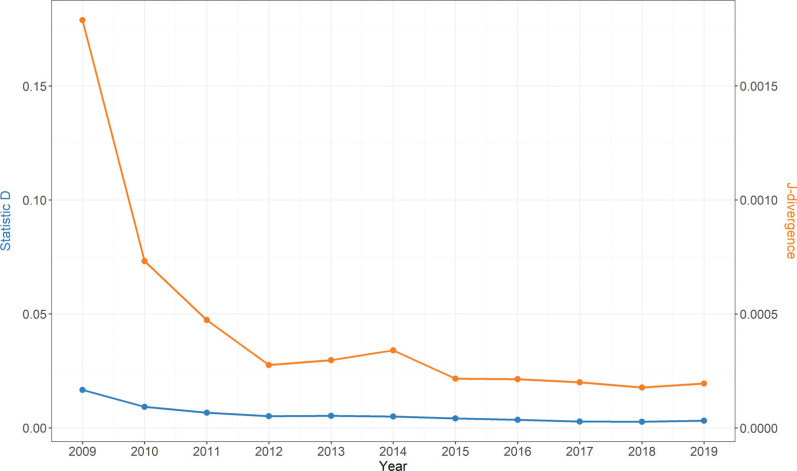
Results of K-S test and J-divergence method.

To compute the J-divergence, the original data and linked data are divided into 100 bins, the 100 bins are created based on the 100 equal intervals of log house price in the original data in a given year. The results of the J-divergence and K-S tests are shown in Fig. 5. The p-values of all the K-S tests are less than 0.05 (the conventional default threshold for statistical significance), indicating a statistically significant difference between the original house price data and the linked house price data. The D statistic is relatively low (less than 0.007) after 2010. This demonstrates that the house price datasets before and after linkage are highly similar after 2010. The J-divergence results also show that the linked data exhibits relatively low information loss after 2010. Given the information lost in terms of J-divergence is slightly higher in 2010 compared to the loss after 2010, the newly created house price data from 2011 to 2019 is more representative than that for other years. Therefore we keep the 2011 to 2019 time period.

#### Linked dataset between 2011 and 2019

There were 7,249,259 full market value transactions in England and Wales between 1/1/2011 and 31/10/2019. Of these 6,753,335 have been successfully linked to EPC records. The overall match rate for this period is 93.15%. To support more advanced understanding of match rate spatially, the National Statistics Postcode Lookup (NSPL) (November 2019 version) is used to geo-reference both the linked data and original pre-linked LR-PPD by postcode to 2011 Census Output Area (OA) code, Lower Layer Super Output Area (LSOA) code and Middle Layer Super Output Area (MSOA) code [23]. Then the ONS hierarchical lookup table [39] is used to relate the OAs with Local authorities (LAs) and Regions information. Twenty-eight linked transactions and 3001 transactions in LR-PPD were lost during this process.

**Figure 6:**
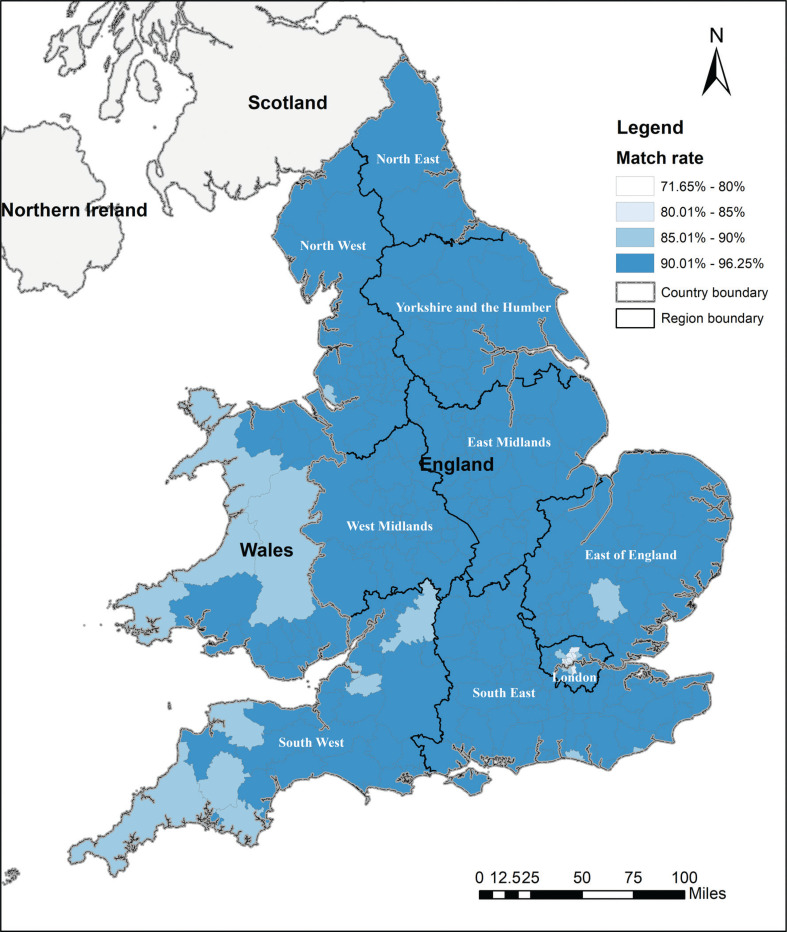
Overall match rates at local authority level between 2011 and 2019.

With the geo-referenced data, the overall match rates between 2011 and 2019 by LA (Fig. 6) are not equally distributed. Ninety-two percent of LAs in England and Wales have a match rate over 90%. Only two LAs (City of London and Isles of Scilly) have a match rate under 80%, these are 71.65% and 76.65%, respectively. The remaining 8% of LAs (26 LAs) show a match rate between 80% and 89.81%.

**Figure 7:**
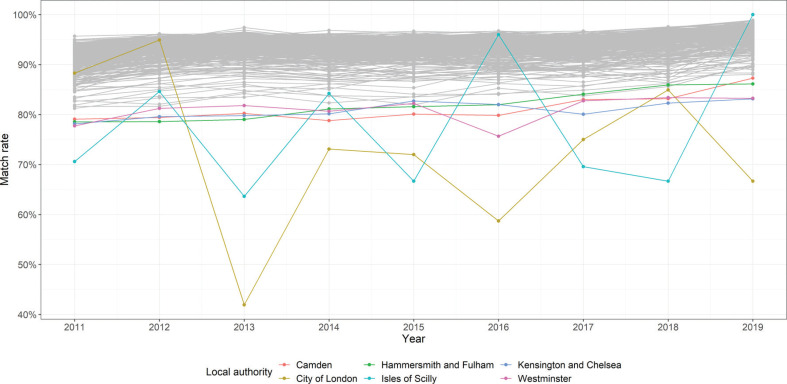
Match rate across local authority in England and Wales, 2011–2019.

Looking at annual match rates across LAs in England and Wales (Fig. 7), 70% of LAs represent an annual match rate over 90% from 2011 to 2019, while 98% of the LAs represent an annual match rate over 80%. Figure 7 colours the six LAs with annual match rates lower than 80%. They are Isles of Scilly, City of London, Camden, Hammersmith and Fulham, Kensington and Chelsea, and Westminster. Only two LAs (City of London and Isles of Scilly), both of which are small in terms of their numbers of transactions, show an obvious fluctuation during this nine-year period. The rates between 2011 and 2019 are, for the remaining 346 LAs, very stable over time with a slight fall after 2015. Overall, the majority of LAs with a high match rate in 2011 maintained a high rate subsequently.

Properties that feature in the LR-PPD (1/1/2011–31/10/2019) are not fully available in Domestic EPCs (1/10/2008–31/8/2019), this is the main reason for unequal match rates across LAs. For 18,980 transactions (2011–2019) relating to 6375 postcode units, Domestic EPCs cannot be found. For example, Domestic EPCs in the City of London at postcode ‘EC2Y 9BB’ are not available hence transactions in ‘EC2Y 9BB’ cannot be successfully matched, 0.26% of house price transactions in the LR-PPD (1/1/2011–31/10/2019) fail to link for this reason. Some transactions in the LR-PPD can relate to a postcode unit which is also identified in the EPC data but contain no matching property identifiers. For example, one flat sold in 2011 in Camden failed to match because Domestic EPCs are not available for this property. The potential reasons for non-availability of property records in Domestic EPCs could be that records have been incorrectly loaded by the surveyor or that the property owner has opted out.

### Data cleaning

Of the linked data, 6,753,307 records can be geo-referenced by linking the NSPL between 1/1/2011 and 31/10/2019 in England and Wales. This data comprises the transaction information in the LR-PPD together with property size (total floor area and number of habitable rooms) in the EPCs. Some properties’ total floor area and number of habitable rooms are recorded in the EPCs with missing or unlikely values (e.g., total floor area records as 0.01). This data is excluded prior to analysis. All the excluded transactions along with cleaning methods are listed in Table 1, which accounts for 15.11% of the linked geo-referenced data.

**Table 1.** List of transactions excluded from the linked geo-referenced data

No.	Method	Transaction count	Proportion of all excluded transactions
1	Transactions where total floor area or number of habitable rooms are NA or 0.	1,016,247	99.59%
2	Transactions where total total floor area is smaller than 9 m^2^ or larger than 974 m^2^.	555	0.05%
3	Transactions where total price per m^2^ is larger than 50,000 £/m^2^ or price per m^2^ is smaller than 200 £/m^2^.	841	0.08%
4	Transactions where floor area per habitable room is larger than 100 m^2^.	887	0.09%
5	Transactions where the number of habitable rooms is larger than 20.	476	0.05%
6	Transactions where floor area per habitable room is smaller than 6.51 m^2^.	1,463	0.14%
Overall		1,020,469	100%

After removing the transactions listed in Table 1, 5,732,838 transactions are left. This represents 79.11% of full market property sales in the LR-PPD in England and Wales between 1/1/2011 and 31/10/2019. This linked dataset, like the LR-PPD, fully covers all the regional areas, local authorities and MSOAs in England and Wales. The LR-PPD covers 99.99% of LSOAs and this is also the same for the final linked data. Although the newly linked data is not as comprehensive as the LR-PPD, it is the largest open access house price dataset in England and Wales (1/1/2011–31/10/2019) containing both the transaction price and total floor area.

## Dataset access

The final linked dataset details 5,732,838 transactions in England and Wales (1/1/2011–31/10/2019). It not only adds in a property’s total floor area and the number of habitable rooms, but also includes a new unique identifier (i.e., id) and other non-address fields (except LMK_KEY field) in the Domestic EPC dataset. Codes for other commonly used spatial units from Output Area (OA) to region are also included in this dataset. It contains 105 fields written in upper or lower case. All the fields written in upper case come from Domestic EPCs, the 33 remaining fields written in lower case are introduced in Github (https://github.com/Bin-Chi/Link-LR-PPD-and-Domestic-EPCs).

The linked, original EPCs and LR-PPD datasets are stored in CSV format and deposited in UKDA ReShare [40]. Postcode and address elements in the linked data stem from address information in LR-PPD, which is subject to Royal Mail copyright. The Royal Mail confirmed on 25/8/2020 that this linked data can be shared both by the first author and by the UK Data Service on the same terms as the original datasets. Therefore the linked data is under a licence that precludes commercial use. Meanwhile, the data linkage is conducted in R and stored in PostGIS. They are also open available in the UKDA ReShare under the same licence [40].

## Potential dataset use and reuse

The newly linked dataset offers directly useable information on house price per square metre along with transaction price, total floor area, number of habitable rooms, transaction date and commonly used geographical area identifiers at and over postcode geographical level in England and Wales. As the LR-PPD data for the most recent two months may be incomplete due to the delay between the property transaction and its registration in Land Registry [21], we suggest researchers use transactions before 31/8/2019. This could support quantitative house price research in terms of house price variation within England and Wales after 2011 at multi-geographical scales over postcode level [41]. It also can be used to explore the relationship between house price and a property’s energy performance [30,31,42]. In addition, as the LR-PPD is updated monthly and the Domestic EPCs are updated two or four times a year, the open access codes will allow for future updates and thereby maintain a continuously updated dataset of residential property prices in England and Wales.

In this paper, we provide three technical validation approaches (section: Technical validation) to inform potential users of the data quality issues associated with different years in the dataset. In Table 1, a series of rules are described which we have used to exclude potential errors in the dataset. These are our suggestions and very reasonably, users could develop their own exclusion criteria for use with the raw linked data. In this dataset, before the data linkage, all transactions designated as category B (Additional Price Paid entry) and other property types are removed. Researchers could add these entries back in by modifying the related code shared via the UK Data Service Reshare service (https://reshare.ukdataservice.ac.uk/854240/). To further benefit non-commercial users who would like to access the latest orginal linkage dataset before the technical validation process, we will annually publish a simple version of the latest raw linkage data via the Greater London Authority (GLA)’s London Datastore.

For users who would like to update the linkage dataset themselves with the linkage code, the Domestic EPCs downloaded may be different from the third version used in this paper. For example, by the time this paper was under open review in February 2021, Domestic EPCs had reached their sixth released version (1/10/2008–20/9/2020). This new version covers more variables than the third version (e.g., building’s construction age band). Moreover, this sixth version has a different sample size of Domestic EPCs for the same time period compared with the third version. The reasons for this difference are complex, although one of the main reasons is that some property owners are withdrawing their EPC records from the publicly available platform. For users who use the latest linked data to explore house prices during the coronavirus pandemic, we highly recommend Neal Hudson’s blog [43] to gain an understanding of how the pandemic increased the HM Land Registry time lag in registrations.

## Conclusions

The linkage method was orignally created to enrich the geo-referenced house price dataset in England before 31/7/2017 [35], it still shows a smiliar performance when updated with new published house prices and covers Wales as shown in this research. Within the linkage, properties in the LR-PPD and Domestic EPC dataset have slightly different names (e.g., ‘CLEATOR STREET’ vs. ‘CLEATER STREET’). We manually correct this type of mismatched address string for the properties located in England and record this correction within the linkage codes. This contributes to a less than 1% increase in the total matching rate. Our futher linkage research is to focus on fixing this issue in Wales and for the newly updated transactions in England.

We expect that this new house price dataset will enable new research directions in UK housing analysis. To date, most hedonic house price models have had to contend with the confounding influence of variations in dwelling size in different housing market areas. This new dataset will enable more parsimonious models of price variation to be explored where proxies for size can be dispensed with.

## Data Availability

The datasets generated during and/or analysed during the current study are available in the repository: https://reshare.ukdataservice.ac.uk/854240/.

## Appendix A

**Table A.** New address variables created from LR-PPD and Domestic EPC datasets for data linkage

Variable	Create method	Dataset
ADD1	Capitalise all strings in ADDRESS1, then remove leading and trailing whitespaces	Domestic EPCs
ADD2	Capitalise all strings in ADDRESS2, then remove leading and trailing whitespaces	Domestic EPCs
ADD3	Capitalise all strings in ADDRESS3, then remove leading and trailing whitespaces	Domestic EPCs
ADD	Capitalise all strings in ADDRESS, then remove leading and trailing whitespaces	Domestic EPCs
ADD2NEW	Delete all ‘-’ in the ADD2	Domestic EPCs
ADDC	Delete all ‘/’, ‘.’, ‘’’ punctuation characters and blank spaces in ADD	Domestic EPCs
ADDU	Delete the ‘UNIT’ string in the ADD, then delete all commas and blank spaces	Domestic EPCs
ADDC3	Delete all commas in ADDC	Domestic EPCs
ADDCC	Delete all ‘-’, ‘/’, ‘.’, ‘’’ punctuation characters and blank spaces in ADD	Domestic EPCs
ADDCCC	Delete all commas in ADDCC	Domestic EPCs
ADDC4	Delete all ‘/’, ‘.’, ‘-’ punctuation characters and blank spaces in ADD	Domestic EPCs
ADDC6	Delete all ‘’’, commas and blank spaces in ADD	Domestic EPCs
ADDRE	Delete all blank spaces in ADD	Domestic EPCs
ADDREC	Delete all commas in ADDRE	Domestic EPCs
ADD1C	Delete all ‘/’, ‘.’, ‘’’ punctuation characters and blank spaces in ADD1	Domestic EPCs
ADD1CC	Delete all ‘-’ punctuation characters in ADD1C	Domestic EPCs
ADD1C2	Delete all commas in ADD1C	Domestic EPCs
ADD1C3	Delete all commas and blank spaces in ADD1	Domestic EPCs
ADD1C6	Delete the ‘UNIT’ in ADD1, then delete all commas and blank spaces	Domestic EPCs
ADD1C4	Delete all ‘’’ punctuation characters in ADD1C3	Domestic EPCs
ADD1C5	Delete all ‘.’ and blank spaces in ADD1	Domestic EPCs
ADD1C7	Delete all commas and blank spaces in ADD1	Domestic EPCs
ADD1C8	Delete all commas in ADD1C5	Domestic EPCs
ADD1C9	Delete all blank spaces in ADD1	Domestic EPCs
ADD1C10	Delete all ‘/’ punctuation characters in ADD1	Domestic EPCs
ADD12C2	Delete all commas in ADD12	Domestic EPCs
ADD12C	Delete all ‘.’, ‘’’, ‘/’ punctuation characters in ADD12	Domestic EPCs
ADD12C1	Delete all ‘.’, ‘’’, ‘/’ punctuation characters and commas in ADD12	Domestic EPCs
ADD12C3	Delete all ‘.’, ‘’’, ‘/’, ‘-’ punctuation characters and commas in the ADD12	Domestic EPCs
ADD12C4	Delete all ‘.’, ‘-’, ‘/’ and blank spaces in ADD12	Domestic EPCs
ADD12C5	Delete all ‘.’, ‘,’ and blank spaces in ADD12	Domestic EPCs
ADD13C	Delete ‘.’, ‘’’, ‘/’ punctuation characters and blank spaces in ADD13	Domestic EPCs
ADD13C1	Delete all commas in ADD13C	Domestic EPCs
ADD13C2	Delete all commas in ADD13	Domestic EPCs
ADD23C	Delete ‘.’, ‘’’, ‘/’ punctuation characters in ADD23	Domestic EPCs
ADD23C1	Delete all commas in ADD23C	Domestic EPCs
ADD161	For the ADD1 containing a comma, select the text before the first comma	Domestic EPCs
ADD161x	Select the text before the first comma in ADD1	Domestic EPCs
ADD162	For the ADD1 containing a comma, select the strings after the first comma	Domestic EPCs
ADD165	For the ADD1 containing a comma and ‘.’ punctuation characters, select the strings after the first comma, then delete the ‘.’ punctuation character	Domestic EPCs
add1sp	If ADD2 does not start with number string and also ADD1 does not contain a word with one character, select the strings before the first blank space in ADD1	Domestic EPCs
add63	Delete all ‘-’ and ‘.’ in ADD162	Domestic EPCs
add1nn	Delete all ‘NO’ strings in ADD1, then delete all commas	Domestic EPCs
ADD1df1	Delete ‘FLAT’ string in ADD1 and then select first word boundary, then delete all commas	Domestic EPCs
ADD1du	Delete the ‘UNIT’ string in ADD1, then delete all commas and blank spaces	Domestic EPCs
ADD163	Select all strings before the first blank space in ADD1	Domestic EPCs
add261	For the ADD2 containing a comma, select all strings before the first comma	Domestic EPCs
add263	Select all strings before the first blank space in ADD2, then delete all commas	Domestic EPCs
add31	Delete ‘’’,‘.’ and ‘/’ in ADD3	Domestic EPCs
fladd1c	Delete all blank spaces in fladd1	Domestic EPCs
fladdc	Delete all commas in the fladd	Domestic EPCs
ADD1dff	If the ADD1 has ‘FLAT’, delete ‘FLAT’ string in ADD1	Domestic EPCs
add264	Select the strings after the first blank space in ADD2	Domestic EPCs
add2641	Select the strings after the first comma in ADD2	Domestic EPCs
apADD1	Delete ‘-’, ‘/’, ‘.’, ‘’’ ‘,’ punctuation characters and blank spaces in apadd1	Domestic EPCs
ADDr61	For the ADD containing a comma, select strings before the first comma	Domestic EPCs
ADDr62	For the ADD containing a comma, select all strings after the first comma, then delete the ‘-’, ‘’’, ‘.’ and ‘/’ punctuation characters	Domestic EPCs
add361	For the ADD3 containing a comma, then select the text before the first comma	Domestic EPCs
ADDC5	Delete all ‘/’, ‘.’ punctuation characters and blank spaces in ADD	Domestic EPCs
ADDC8	Delete all ‘.’,‘’’ punctuation characters and blank spaces in ADD	Domestic EPCs
ADDC9	Delete all ‘.’,‘’’ and ‘/’ punctuation characters in ADD	Domestic EPCs
ADDC10	Delete all ‘-’, ‘/’, ‘.’, ‘’’, ‘,’ punctuation characters and blank spaces in ADD	Domestic EPCs
ADD262	For the ADD2 containing a comma, then select all strings after the first comma	Domestic EPCs
add1f61	If the ADD1 in EPC data has ‘FLAT’ string, delete the ‘FLAT’ string, then keep all strings before the first blank space, and then delete all commas	Domestic EPCs
add1f61f2	Combine ‘FLAT’ and add1f61 with a blank space, then combine ADD2 with a comma and a blank space, then delete all blank spaces and commas	Domestic EPCs
add1f61f3	Combine ‘FLAT’ and add1f61 with a blank space, then combine ADD2 with a comma and a blank space, then delete all blank spaces	Domestic EPCs
adddap	Delete the ‘APARTMENT’ string in ADD, then delete all blank spaces	Domestic EPCs
saonn	Delete all ‘/’ punctuation characters in SAON	LR-PPD
paonn	Delete all ‘’’, ‘.’ punctuation characters in PAON	LR-PPD
paonn2	Delete all commas and blank spaces in PAON	LR-PPD
paonn3	Delete all ‘-’ and blank spaces in paonn	LR-PPD
streetn	Delete all ‘’’ punctuation characters in street	LR-PPD
streetn1	Delete ‘-’, ‘.’, ‘’’ punctuation characters and blank spaces in street	LR-PPD
streetn2	Delete ‘-’, ‘’’ punctuation characters and blank spaces in street	LR-PPD
streetn5	Delete ‘/’, ‘.’, ‘’’ punctuation characters in street	LR-PPD
localityn	Delete all ‘’’, ‘.’ punctuation characters in locality	LR-PPD
saonpaonstreet31	Delete all commas in saonpaonstreet3	LR-PPD
saonpaonstreetn31	Delete all commas in saonpaonstreetn3	LR-PPD
paon61	For the PAON containing comma, grab the strings before the first comma	LR-PPD
paon61c	Delete all blank spaces in paon61	LR-PPD
paon61x	Select the strings before the first comma	LR-PPD
paon62	For the PAON containing a comma, subset the strings after the first comma	LR-PPD
paon62c	Subset the strings after the first comma in PAON	LR-PPD
saonpaon62cstreetn2	Combine SAON and paon62c with a comma and a blank space, then combine streetn with a blank space, then delete all blank spaces	LR-PPD
saonpaon62cstreetn	Combine SAON and paon62c with a blank space, then combine streetn with a blank space, then delete all blank spaces	LR-PPD
saonpaon62cstreet	Combine SAON and paon62c with a comma and a blank space, then combine street with a comma and a blank space, then delete all blank spaces	LR-PPD
paon64	Subset the string before the first blank space in PAON	LR-PPD
paon641	Subset the string after the first blank space in PAON	LR-PPD
paon65	For the PAON containing a comma, extract the last word from PAON	LR-PPD
paon65n	For the paonn containing a comma, extract the last word from paonn	LR-PPD
saon2	Delete ‘APARTMENT’ string in SAON	LR-PPD
fldsaon	If SAON contains ‘FLAT’ string and PAON does not start with number string. Then delete ‘FLAT’ string in SAON	LR-PPD
fldsaon1	If SAON contains ‘FLAT’ string and PAON starts with number string. Then delete ‘FLAT’ string in SAON	LR-PPD
saon7	Replace ‘FLAT’ string by ‘APARTMENT’ string in SAON	LR-PPD
saon71	Replace ‘FLAT’ string by ‘APARTMENT’ string in saonn	LR-PPD
saonn4	Delete ‘FLAT’ string in saonn	LR-PPD
saon1	Replace ‘APARTMENT’ string by ‘FLAT’ string in saonn	LR-PPD
saonn2	Delete ‘APARTMENT’ string in saonn	LR-PPD
saonn3	Delete ‘.’ and ‘/’ in SAON	LR-PPD
ADD1num	Extract the number string in ADD1	LR-PPD
saonn5	If the SAON contains ‘APARTMENT’, replace ‘APARTMENT’ string by ‘UNIT’ string in SAON and then delete ‘/’ punctuation characters	LR-PPD
sao1	Replace ‘APARTMENT’ string by ‘FLAT’ string in SAON	LR-PPD
saon8	If SAON contains the ‘LOFT’ string, replace ‘LOFT’ by ‘FLAT’	LR-PPD
saon4	Delete ‘FLAT’ string in SAON	LR-PPD
paon6164	Select the number string from paon61	LR-PPD
paon6163	Select all non-digitals from paon61	LR-PPD
paon11	Delete all comma in the PAON	LR-PPD
ADD12	Combine ADD1 and ADD2 with a comma and a blank space, then delete all blank spaces	Domestic EPCs
ADD12C6	Combine ADD1 and ADD2 with a blank space, then delete all blank spaces	Domestic EPCs
ADD12new	Combine ADD1 and ADD2NEW with a blank space, then delete all ‘/’, ‘.’, ‘’’ punctuation characters, blank spaces and commas	Domestic EPCs
ADD13	Combine ADD1 and ADD3 with a comma and a blank space, then delete all blank spaces	Domestic EPCs
ADD23	Combine ADD2 and ADD3 with a blank space, then delete all blank spaces	Domestic EPCs
ADD66	For ADD162 containing ‘-’ punctuation characters, delete ‘-’ in ADD162, then combine ADD161 and ADD162 with a comma and a blank space	Domestic EPCs
ADD662	Combine ADD66 and ADD2 with a comma and a blank space, then delete all commas and blank spaces	Domestic EPCs
ADD67	Combine ADD161 and ADD165 with a comma and a blank space, then delete all commas and blank spaces	Domestic EPCs
ADDSP12	Combine add1sp and add2 with a comma and a blank space, then delete all commas and blank spaces	Domestic EPCs
ADD68	Combine add161 and add63 with a comma and a blank space, then delete all ‘’’ and blank spaces	Domestic EPCs
ADD69	Combine add1nn and ADD2 with a comma and a blank space, then delete all blank spaces	Domestic EPCs
ADD1632	Combine ADD163 and ADD2 with a blank space, then delete all commas and blank spaces	Domestic EPCs
flADD	Combine ‘FLAT’ string and ADD with a comma and a blank space, then delete all commas and blank spaces	Domestic EPCs
ADD2611	Combine add261 and add1 with a comma and a blank space, then delete all commas and blank spaces	Domestic EPCs
fladd1	Combine ‘FLAT’ and ADD1 with a blank space	Domestic EPCs
fladd	Combine ‘FLAT’ and ADD with a blank space, then delete all blank spaces	Domestic EPCs
flADD13	Combine fladd1 and add31 with a blank space, then delete all commas and blank spaces	Domestic EPCs
ADD5	Combine add263 and ADD1dff, then combine add264, then delete all blank spaces	Domestic EPCs
apadd1	Combine ‘APARTMENT’ and ADD1 with a blank space	Domestic EPCs
ADDr66	Combine ADDr61 and ADDr62 with a comma and a blank space, then delete all commas and blank spaces	Domestic EPCs
ADD6	Combine ADD1 and ADD2 with a comma and a blank space, then combine add361 with a comma and a blank space, then delete all ‘/’, ‘.’, ‘’’ punctuation characters and blank spaces	Domestic EPCs
add12643	Combine ADD1 and add264 with a comma and a blank space, then combine ADD3 with a comma and a blank space, then delete all blank spaces	Domestic EPCs
ADD1264	Combine ADD1 and add2641 with a comma and a blank space, then delete all blank spaces and commas	Domestic EPCs
ADD1265	Combine ADD1 and add264 with a comma and a blank space, then delete all blank spaces	Domestic EPCs
ADD8	Combine ADD1C10 and ADD2 with a comma and a blank space, then delete all blank spaces	Domestic EPCs
ADD7	Combine ADD161x and ADD2 with a blank space, then delete all blank spaces	Domestic EPCs
ADD1num2	Combine ADD1num and ADD2 with a comma and a blank space, then delete, ‘/’, ‘.’, ‘’’ punctuation characters and all blank spaces	Domestic EPCs
ADD1262	Combine ADD1 and ADD262 with a comma and a blank space, then delete all blank spaces	Domestic EPCs
ADD1263	Combine ADD1 and ADD2641 with a comma and a blank space, then delete all blank spaces	Domestic EPCs
ADD1262C	Combine ADD1 and ADD262 with a comma and a blank space, then delete all blank spaces and commas	Domestic EPCs
ADD1262cc	Combine ADD1 and ADD262 with a comma and a blank space, then delete all blank spaces and ‘’’	Domestic EPCs
apadd1632	Combine ‘APARTMENT’ and add163 with a blank space, then combine with ADD2 with a comma and a blank space, then delete all blank spaces and commas	Domestic EPCs
saonpaonstreet	Combine SAON and PAON with a comma and a blank space, then combine street with a blank space, then delete all blank spaces	LR-PPD
saonpaonstreet5	Combine SAON and PAON with a comma and a blank space, then combine street with a blank space, then delete all blank spaces and commas	LR-PPD
saonpaonstreet1	Combine SAON and PAON with a comma and a blank space, then combine street with a comma and a blank space, then delete all blank spaces	LR-PPD
saonpaonstreet2	Combine SAON and PAON with a blank space and then remove leading and trailing whitespaces, then combine street with a comma and a blank space, then delete all blank spaces	LR-PPD
saonpaonstreetn	Combine saonn and paonn with a comma and a blank space, then combine streetn with a blank space, then delete all blank spaces	LR-PPD
saonpaonstreetn1	Combine saonn and paonn with a comma and a blank space, then combine streetn with a comma and a blank space, then delete all blank spaces	LR-PPD
saonpaonstreetn2	Combine saonn and paonn with a blank space, then combine streetn with a comma and a blank space, then delete all blank spaces	LR-PPD
saonpaonlo	Combine SAON and PAON with a blank space, then combine locality with a comma and a blank space, then delete all blank spaces	LR-PPD
saonpaonlon	Combine saonn and paonn with a blank space, then combine localityn with a comma and a blank space, then delete all blank spaces	LR-PPD
saonpaonstreet3	Combine SAON and PAON with a blank space and then remove leading and trailing whitespaces, then delete combine street with a blank space, then delete all blank spaces	LR-PPD
saonpaonstreetn3	Combine saonn and paonn with a blank space, then delete combine streetn with a blank space and then remove leading and trailing whitespaces, then delete all blank spaces	LR-PPD
saonpaonstreetlo	Combine SAON and PAON with a comma and a blank space, then combine street with a comma and a blank space and then remove the leading and trailing whitespaces, then combine locality with a comma and a blank space, then delete all blank spaces	LR-PPD
saonpaonstreetnlo	Combine saonn and paonn with a comma and a blank space, then combine streetn with a comma and a blank space and then remove the leading and trailing whitespaces, then combine localityn with a comma and a blank space, then delete all blank spaces	LR-PPD
saonpaon1	Combine SAON and PAON with a blank space, then delete all blank spaces	LR-PPD
saonpaon2	Combine SAON and PAON with a comma and a blank space, then delete all blank space and all blank spaces	LR-PPD
saonpaon3	Combine SAON and PAON with a comma and a blank space	LR-PPD
paonstreetlo	Combine PAON and street with a comma and a blank space, then combine locality with a comma and a blank space, then delete all blank spaces	LR-PPD
paonstreetnlo	Combine paonn and streetn with a comma and a blank space, then combine localityn with a comma and a blank space, then delete all blank spaces	LR-PPD
paonstreetlo1	Combine PAON and street with a blank space, then combine locality with a comma and a blank space, then delete all blank spaces	LR-PPD
paonstreetnlo1	Combine paonn and streetn with a blank space, then combine localityn with a comma and a blank space, then delete all blank spaces	LR-PPD
paonstreetlo2	Combine PAON and street with a blank space, then combine locality with a blank space, then delete all blank spaces and commas	LR-PPD
paonstreetn	Combine paonn and streetn with a comma and a blank space, then delete all blank spaces	LR-PPD
paon66	Combine paon62 and paon61 with a comma and a blank space, then delete all blank spaces	LR-PPD
paon65streetlo	Combine paon65 and street with a comma and a blank space, then combine locality with a comma and a blank space, then delete all blank spaces	LR-PPD
paon65streetnlo	Combine paon65n and streetn with a comma and a blank space, then combine localityn with a comma and a blank space, then delete all blank spaces	LR-PPD
paon65streetlo1	Combine paon65 and street with a blank space, then combine locality with a blank space, then delete all blank spaces and commas	LR-PPD
paon61streetlo	Combine paon61 and street with a comma and a blank space, then combine locality with a comma and a blank space, then delete all blank spaces	LR-PPD
paon61streetlo1	Combine paon61 and street with a blank space, then combine locality with a blank space, then delete all blank spaces and commas	LR-PPD
paon61lo	Combine paon61 and locality with a comma and a blank space, then delete all blank spaces	LR-PPD
paon61street	Combine paon61 and street with a blank space, then delete all blank spaces and commas	LR-PPD
paon65street	Combine paon65 and street with a blank space, then delete all blank spaces and commas	LR-PPD
paon66streetlo	Combine paon62 and paon61 with a blank space, then combine street with a blank space, then combine locality with a blank space, then delete all commas and blank spaces	LR-PPD
paon61new	Combine ‘THE’ and paon61 with a blank space	LR-PPD
paonstreetlo3	Combine PAON and street with a comma and a blank space, then combine locality with a comma and a blank space, then delete all blank spaces and commas	LR-PPD
paonstreet	Combine PAON and street with a comma and a blank space, then delete all commas and blank spaces	LR-PPD
paonstreetn1	Combine PAON and streetn1 with a comma and a blank space, then delete all commas and blank spaces	LR-PPD
paonstreet1	Combine PAON and street with a comma and a blank space, then delete all blank spaces	LR-PPD
paonstreet2	Combine PAON and street with a blank space, then delete all blank spaces	LR-PPD
paon62streetlo	Combine paon62 and street with a comma and a blank space, then combine locality with a comma and a blank space, then delete all blank spaces	LR-PPD
paon62streetlo1	Combine paon62 and street with a blank space, then combine locality with a blank space, then delete all blank spaces and commas	LR-PPD
paonflat	Combine ‘FLAT’ string and PAON with a blank space	LR-PPD
paonfstreet	Combine paonflat with street with a comma and a blank space, then delete all blank spaces	LR-PPD
paonap	Combine ‘APARTMENT’ string and PAON with a blank space	LR-PPD
paonapstreet	Combine paonap with street with a comma and a blank space, then delete all blank spaces	LR-PPD
paonfstreet1	Combine paonflat with street with a blank space, then delete all blank spaces	LR-PPD
paonfstreetn5	Combine paonflat with streetn5 with a blank space, then delete all blank spaces	LR-PPD
paonstreet3	Combine PAON and street with a blank space, then delete all blank spaces and commas	LR-PPD
paonapstreet1	Combine paonap with street with a blank space, then delete all blank spaces	LR-PPD
paonapstreet2	Combine paonap with street with a blank space, then delete all blank spaces and commas	LR-PPD
paonapstreetn5	Combine paonap with streetn5 with a blank space, then delete all blank spaces	LR-PPD
paonstreet4	Replace ‘FLAT’ by ‘APARTMENT’ in paonstreet3	LR-PPD
paonfl1	Combine ‘FLAT,’ string and strings in PAON with a blank space	LR-PPD
paonf1streetn5	Combine paonfl1 with streetn5 with a comma and a blank space, then delete all blank spaces	LR-PPD
paonfstreetn6	Combine paonflat with streetn5 with a comma and a blank space, then delete all blank spaces	LR-PPD
flpaon3streetn5	Combine ‘FLAT’ string and PAON with a blank space, then combine with streetn5 with a blank space then delete all blank space and ‘-’ punctuation characters	LR-PPD
saonpaon65street	Combine SAON and paon65 with a comma and a blank space, then combine street with a comma and a blank space, then delete all blank spaces	LR-PPD
saonpaon62streetn2	Combine SAON and paon62 with a comma and a blank space, then combine streetn with a blank space, then delete all blank spaces	LR-PPD
saonpaon61street	Combine SAON and paon61 with a blank space, then combine street with a comma and a blank space, then delete all blank spaces and commas	LR-PPD
saonpaon61xstreet	Combine SAON and paon61x with a blank space, then combine street with a comma and a blank space, then delete all blank spaces and commas	LR-PPD
saonpaonn	Combine saonn and paonn with a comma and a blank space, then delete all blank spaces	LR-PPD
saon2street	Combine saon2 and street with a comma and a blank space, then delete all blank spaces	LR-PPD
saon2paon61street	Combine saon2 and paon61 with a blank space, then combine street with a comma and blank space, then delete all blank spaces	LR-PPD
flsaonpaonstreet0	Combine flsaon and PAON with a comma and a blank space and then combine street with a comma and a blank space	LR-PPD
flsaonpaon1	Combine flsaon and PAON with a blank space, then delete all blank spaces	LR-PPD
flsaonpaon2	Combine flsaon and PAON with a comma and a blank space, then delete all blank spaces	LR-PPD
flsaonpaon3	Combine flsaon3 and PAON with a comma and a blank space, then delete all blank spaces	LR-PPD
flsaon	For the SAON starts with number string, combine ‘FLAT’ string with SAON with a blank space	LR-PPD
flsaon1	For the SAON starts with number string, combine ‘FLAT’ string with saonn with a blank space	LR-PPD
flsaon3	Combine ‘FLAT’ string with SAON with a blank space	LR-PPD
flsaon1paonstreetn2	Combine flsaon1 with paonn with a comma and a blank space, then combine the streetn2 with a comma and a blank space, then delete all blank spaces	LR-PPD
flsaonpaonstreet1	Combine flsaon with PAON with a blank space, then combine the street with a blank space, then delete all blank spaces and commas	LR-PPD
flsaonpaon62street1	Combine flsaon and paon62 with a blank space, then combine street with a blank space, then delete all blank spaces and commas	LR-PPD
fldsaonpaonstreet1	Combine fldsaon and PAON with a blank space, then combine street with a blank space, then delete all blank spaces and commas	LR-PPD
saon7paonstreet1	Combine saon7 and PAON with a comma and a blank space, then combine street with a blank space, then delete all blank spaces	LR-PPD
saon7paonstreet2	Combine saon7 and PAON with a blank space, then combine street with a blank space, then delete all blank spaces and commas	LR-PPD
apsaon	For SAON starts with number string, combine ‘APARTMENT’ string with SAON with a blank space	LR-PPD
apsaonpaonstreet1	Combine apsaon and PAON with a blank space, then combine street with a blank space, then delete all blank spaces and commas	LR-PPD
saon7paonstreetn	Combine saon71 and paonn with a comma and a blank space, then combine streetn with a blank space, then delete all blank spaces	LR-PPD
saon7paonn	Combine saon7 and paonn with a comma and a blank space, then delete all blank spaces	LR-PPD
saon7paon	Combine saon7 and PAON with a comma and a blank space, then delete all blank spaces	LR-PPD
saon4paonstreetn	Combine saonn4 and paonn with a comma and a blank space, then combine streetn with a blank space, then delete all blank spaces	LR-PPD
saon4paonstreetn1	Combine saonn4 and paonn with a blank space, then combine streetn with a comma and a blank space, then delete all blank spaces	LR-PPD
apsaonpaon6streetn	Combine apsaon and paon62 with a comma and a blank space, then combine streetn with a blank space, then delete all blank spaces	LR-PPD
flsaonpaonstreetn	Combine ‘FLAT’ string with SAON with a blank space, then combine paonn with a comma and a blank space, then combine with streetn with a blank space, then delete all blank spaces	LR-PPD
saon4paonstreetn3	Combine saonn4 and paonn with a blank space, then combine streetn with a blank space, then delete all blank spaces	LR-PPD
saon4paonstreetn4	Combine saonn4 and paonn with a comma and a blank space, then combine streetn with a comma and a blank space, then delete all blank spaces	LR-PPD
saon1paonstreetn	Combine saon1 and paonn with a blank space, then combine streetn with a comma and a blank space, then delete all blank spaces	LR-PPD
saon1paonstreetn1	Combine saon1 and paonn with a comma and a blank space, then combine streetn with a comma and a blank space, then delete all blank spaces	LR-PPD
saon1paonstreetn2	Combine saon1 and paonn with a blank space, then combine streetn with a blank space, then delete all blank spaces and commas	LR-PPD
saon2paonstreetn3	Combine saonn2 and paonn with a blank space, then combine streetn with a blank space, then delete all blank spaces	LR-PPD
saon2paonstreetn2	Combine saonn2 and paonn with a blank space, then combine streetn with a comma and a blank space, then delete all blank spaces	LR-PPD
saonn2paonn1	Combine saonn2 and paonn with a blank space, then delete all blank spaces	LR-PPD
saonpaon62street	Combine SAON and paon62 with a comma and a blank space, then combine street with a blank space, then delete all blank spaces	LR-PPD
saon2paonstreetn	Combine saonn2 and paonn with a comma and a blank space, then combine street with a blank space, then delete all blank spaces	LR-PPD
saonn3paonnstreet	Combine saonn3 and paonn with a comma and a blank space, then combine street with a blank space, then delete all blank spaces	LR-PPD
saonn2paonn1streetn	Combine saonn2 and paonn with a comma and a blank space, then combine street with a blank space, then delete all blank spaces	LR-PPD
saonpaon62streetn1	Combine SAON and paon62c with a comma and a blank space, then combine streetn with a comma and a blank space, then delete all blank spaces	LR-PPD
saon1paonstreet6n	Combine saon1 and paon62c with a comma and a blank space, then combine streetn with a comma and a blank space, then delete all blank spaces	LR-PPD
saon1paonstreet6n1	Combine saon1 and paon62 with a comma and a blank space, then combine street with a blank space, then delete all blank spaces	LR-PPD
saon2paonstreetn4	Combine saonn2 and paonn with a comma and a blank space, then combine streetn with a comma and a blank space, then delete all blank spaces	LR-PPD
saon5paonstreetn1	Combine saonn5 and paonn with a blank space, then combine streetn with a comma and a blank space, then delete all blank spaces	LR-PPD
paonsaon2streetn	Combine paonn and saonn2 with a blank space, then combine streetn with a blank space, then delete all blank spaces	LR-PPD
paon62saonpstreet	Combine paon62 and SAON with a blank space, then combine paon61 with a blank space and then combine street with a comma and a blank space, then delete all blank spaces and commas	LR-PPD
saonpaon66street	Combine SAON and paon62 with a comma and a blank space, then combine paon61 with a blank space, then combine street with a blank space, then delete all blank spaces and commas	LR-PPD
saon1paonstreetn3	Combine saon1 and paonn with a comma and a blank space, then combine streetn with a blank space, then delete all blank spaces	LR-PPD
saon1paonstreet	Combine sao1 and PAON with a comma and a blank space, then combine street with a blank space, then delete all blank spaces	LR-PPD
saon2paonlo	Combine saon2 and PAON with a blank space, then combine locality with a comma and a blank space, then delete all blank spaces	LR-PPD
saon1paon	Combine sao1 and PAON with a comma and a blank space, then delete all blank spaces	LR-PPD
saon1paon61street	Combine sao1 and paon61c with a blank space, then combine street with a comma and a blank space, then delete all blank spaces	LR-PPD
saon1paon1	Combine sao1 and PAON with a blank space, then delete all blank spaces	LR-PPD
psaonpaonstreet	Combine paon64 and SAON, then combine paon641 with a blank space, then combine street with a comma and a blank space, then delete all blank spaces and commas	LR-PPD
saon2paon62street	Combine saon2 and paon62 with a comma and a blank space, then combine street with a comma and a blank space, then delete all blank spaces	LR-PPD
saon2paonstreet	Combine saon2 and PAON with a blank space, then combine street with a comma and a blank space, then delete all blank spaces	LR-PPD
flsaonpaonstreet	Combine flsaon with PAON with a comma and a blank space, then combine the street with a comma and a blank space, then delete all blank spaces and commas	LR-PPD
psaon8street	Combine PAON and fldsaon1, then combine street with a blank space then delete all the blank spaces and commas	LR-PPD
saonstreet	Combine SAON and street with a comma and a blank space, then delete all blank spaces	LR-PPD
saonstreet1	Combine SAON and street with a blank space, then delete all blank spaces and commas	LR-PPD
saonstreet2	Combine SAON and street with a comma and a blank space, then delete all blank spaces and commas	LR-PPD
saonstreet3	Combine SAON and street with a blank space, then delete all blank spaces	LR-PPD
saonstreetlo	Combine SAON and street with a comma and a blank space, then combine with locality with a comma and a blank space, then delete all blank spaces	LR-PPD
unsaonpaonstreet2	Combine ‘UNIT’ string with SAON with a blank space, then combing PAON with a blank space, then combine with street with a comma and a blank space and then delete all blank spaces	LR-PPD
flsaonpaonstreet2	Combine flsaon3 with PAON with a blank space, then combine the street with a comma and a blank space, then delete all blank spaces	LR-PPD
saon7paon6street	Combine saon7 and paon62 with a comma and a blank space, then combine street with a blank space, then delete all blank spaces	LR-PPD
saon8paonstreet2	Combine saon8 and PAON with a blank space, then combine street with a comma and a blank space, then delete all blank spaces	LR-PPD
paonlo	For PAON start with number string, combine PAON and locality with a comma and a blank space, then delete all blank spaces	LR-PPD
flsaonpaonstreet3	Combine flsaon3 with PAON with a blank space, then combine the street with a comma and a blank space, then delete all blank spaces and commas	LR-PPD
saonpaon62steet	Combine SAON and paon62 with a comma and a blank space, then combine street with a comma and blank space, then delete all blank spaces	LR-PPD
flsaonpaon61street	Combine flsaon with paon61 with a blank space, then combine the street with a comma and a blank space, then delete all blank spaces and commas	LR-PPD
flsaonpaon61street1	Combine flsaon with paon61 with a blank space, then combine the street with a comma and a blank space, then delete all blank spaces	LR-PPD
saon4paonstreet	Combine saon4 with PAON with a blank space, then combine the street with a blank space, then delete all blank spaces	LR-PPD
saonpaon61street1	Combine SAON and paon61 with a blank space, then combine street with a comma and a blank space, then delete all blank spaces	LR-PPD
flsaonpaonstreet4	Combine flsaon3 with PAON with a comma and a blank space, then combine the street with a comma and a blank space, then delete all blank spaces	LR-PPD
paonsaonstreet	Combine PAON and SAON, then combine street with a comma and a blank space, then delete all blank spaces	LR-PPD
saonpaon61	Combine SAON and paon61 with a comma and a blank space, then delete all blank spaces	LR-PPD
paonsaonstreet1	Combine PAON and SAON with a comma and a blank space, then combine street with a comma and a blank space, then delete all blank spaces	LR-PPD
apsaonpaon	Combine apsaon and PAON with a blank space, then delete all blank spaces	LR-PPD
saon1paon62street	Combine sao1 and paon62 with a comma and a blank space, then combine street with a comma and a blank space, then delete all blank spaces	LR-PPD
apsaonpaon62street1	Combine apsaon and paon62 with a comma and a blank space, then combine street with a blank space, then delete all blank spaces	LR-PPD
saon2paonstreet1	Combine saon2 and PAON with a blank space, then combine street with a comma and a blank spaces	LR-PPD
apsaonpaonstreet2	Combine apsaon and PAON with a blank space, then combine street with a comma and a blank space, then delete all blank spaces	LR-PPD
psaonpstreet	Combine paon6164 and SAON, then combine paon6163 with a blank space, then combine paon62 with a comma and then combine street with a comma and a blank space and delete all blank spaces	LR-PPD
saonpaonstreet11	Combine SAON and paon11 with a blank space, then combine street with a blank space, then delete all blank spaces	LR-PPD
saonpaon65street1	Combine SAON and paon65 with a comma and a blank space, then combine street with a blank space, then delete all blank spaces	LR-PPD

## Appendix B

**Table B.** Details of matching rules in four stages

Stage No.	Matching rule No.	Matching rule^1^
Stage 1	Matching rule 1	saonpaonstreet=ADDRE
	Matching rule 2	saonpaonstreet1=ADDRE
	Matching rule 3	saonpaonstreet2=ADDRE
	Matching rule 4	saonpaonstreetn=ADDC
	Matching rule 5	saonpaonstreetn1=ADDC
	Matching rule 6	saonpaonstreetn2=ADDC
	Matching rule 7	saonpaonlo=ADDRE
	Matching rule 8	saonpaonlon=ADDC
	Matching rule 9	saonpaonlon=ADDCC
	Matching rule 10	saonpaonstreet=ADD12
	Matching rule 11	saonpaonstreet1=ADD12
	Matching rule 12	saonpaonstreet2=ADD12
	Matching rule 13	saonpaonstreetn=ADD12C
	Matching rule 14	saonpaonstreetn1=ADD12C
	Matching rule 15	saonpaonstreetn2=ADD12C
	Matching rule 16	saonpaonstreet3=ADD12
	Matching rule 17	saonpaonstreetn3=ADD12C1
	Matching rule 18	saonpaonstreetlo=ADDRE
	Matching rule 19	saonpaonstreetnlo=ADDC
	Matching rule 20	saonpaonstreetlo=ADD12
	Matching rule 21	saonpaonstreet3=ADDRE
	Matching rule 22	saonpaonstreetn3=ADDC
	Matching rule 23	saonpaonlo=ADD12
	Matching rule 24	saonpaonlon=ADD12C
	Matching rule 25	saonpaon1=ADDRE
	Matching rule 26	saonpaonstreet31=ADDREC
	Matching rule 27	saonpaonstreetn31=ADDC3
Stage 2	Matching rule 28	paonstreetlo=ADDRE
	Matching rule 29	paonstreetnlo=ADDC
	Matching rule 30	paonstreetlo=ADD12
	Matching rule 31	paonstreetnlo=ADD12C
	Matching rule 32	paonstreetlo1=ADDRE
	Matching rule 33	paonstreetnlo1=ADDC
	Matching rule 34	paonstreetlo1=ADD12
	Matching rule 35	paonstreetnlo1=ADD12C
	Matching rule 36	paonstreetlo2=ADD12C2
	Matching rule 37	paonstreetlo2=ADDREC
	Matching rule 38	paonstreetn=ADD12C3
	Matching rule 39	For the street is null, paonn3=ADD1CC
	Matching rule 40	paon66=ADD1CC
Stage 3	Matching rule 41	paon65streetlo=ADDRE
	Matching rule 42	paon65streetlo=ADD12
	Matching rule 43	paon65streetnlo=ADDCC
	Matching rule 44	paon65streetlo1=ADDREC
	Matching rule 45	paon61streetlo=ADDC
	Matching rule 46	paon61streetlo1=ADDREC
	Matching rule 47	paon61streetlo1=ADDC3
	Matching rule 48	paon61streetlo1=ADD12C1
	Matching rule 49	paon61lo=ADD12C
	Matching rule 50	paon61street=ADD12C1
	Matching rule 51	paon61street=ADD13C1
	Matching rule 52	paon65street=ADDC3
	Matching rule 53	paon65street=ADD1C2
	Matching rule 54	paon66streetlo=ADDCCC
	Matching rule 55	paon66streetlo=ADD12C3
	Matching rule 56	paon65streetlo1=ADD23C1
	Matching rule 57	paon61new=ADD1
	Matching rule 58	paonstreetlo3=ADD12new
	Matching rule 59	paonstreetlo3=ADD13C1
	Matching rule 60	paonstreetlo3=ADD13C2
	Matching rule 61	paonstreet=ADD1C3
	Matching rule 62	PAON=ADD1
	Matching rule 63	paonstreetlo3=ADD662
	Matching rule 64	paonstreet=ADD67
	Matching rule 65	If street in PPD is not null, then paonstreet=ADDSP12
	Matching rule 66	paonstreetn1=ADD1C4
	Matching rule 67	paonstreet=ADDU
	Matching rule 68	paonstreet1=ADD68
	Matching rule 69	paonstreet1=ADD69
	Matching rule 70	Having corrected the mismatched address strings in ADD1 in EPC dataset, then paonstreet1=ADD1C5
	Matching rule 71	Having corrected the mismatched address strings in ADD1 in EPC dataset, then paonstreet2=ADD1C5
	Matching rule 72	Having corrected the mismatched address strings in ADD1 in EPC dataset, then paonn2=ADD1C6
	Matching rule 73	Having corrected the mismatched address strings in ADD1 in EPC dataset, if SAON in PPD is null and ADD in EPCs does not contain a hyphenated number string then , paonstreet3=ADDCCC
	Matching rule 74	If paon61 does not contain ‘FLAT’, ‘FLOOR’ and number strings in PPD data, then paon62streetlo=ADDRE
	Matching rule 75	If paon61 does not contain ‘FLAT’, ‘FLOOR’ and number strings in PPD data, then paon62streetlo=ADD12
	Matching rule 76	If paon61 does not contain ‘FLAT’, ‘FLOOR’ and number strings in PPD data, then paon65streetnlo=ADDCC
	Matching rule 77	If paon61 does not contain ‘FLAT’, ‘FLOOR’ and number strings in PPD data, then paon62streetlo1=ADDREC
	Matching rule 78	paon61streetlo=ADDC
	Matching rule 79	paon61streetlo1=ADDREC
	Matching rule 80	paon61streetlo1=ADDC3
	Matching rule 81	paon61streetlo1=ADD12C1
	Matching rule 82	paon61street=ADD13C1
	Matching rule 83	paon66streetlo=ADDCCC
	Matching rule 84	paon66streetlo=ADD12C3
	Matching rule 85	paonfstreet=ADDRE
	Matching rule 86	paonfstreet=ADD12
	Matching rule 87	paonapstreet=ADDRE
	Matching rule 88	paonfstreet1=ADDRE
	Matching rule 89	paonstreet=ADD1C7
	Matching rule 90	paonstreetn1=ADD1C7
	Matching rule 91	paonstreetn1=ADD1C8
	Matching rule 92	paonstreet1=ADD1C5
	Matching rule 93	paonstreet2=ADD1C5
	Matching rule 94	If the transactions in SE5 7QS, then PAON=ADD1df1
	Matching rule 95	Having corrected the mismatch strings in add1 in EPCs, thenpaonn2=ADD1du
	Matching rule 96	Having corrected the mismatch strings in add1 in EPCs, thenpaon61c=ADD1C9
	Matching rule 97	Having corrected the mismatch strings in add1 in EPCs, if PAON starts with number characters, then paonfstreet1=ADD1C9
	Matching rule 98	If PAON starts with number characters, then paonfstreetn5=ADD1C
	Matching rule 99	paonstreet3=ADD1632
	Matching rule 100	If PAON in PPD starts with number characters and ADD2 in EPC does not start with number characters, then paonapstreet1=ADD12C2
	Matching rule 101	If PAON in PPD starts with number characters, paonapstreetn5=ADD12C1
	Matching rule 102	paonn2=ADDC3
	Matching rule 103	paonstreet3=flADD
	Matching rule 104	paonn2=ADD2611
	Matching rule 105	paonstreet3=flADD13
	Matching rule 106	paonstreet3=ADD13C2
	Matching rule 107	paonstreet4=ADDC3
	Matching rule 108	If PAON in PPD starts with number characters, paonfstreetn5=ADD1C2
	Matching rule 109	paonapstreet2=ADD12C2
	Matching rule 110	paonn2=ADD1C2
	Matching rule 111	paonf1streetn5=ADD12C
	Matching rule 112	paonfstreetn6=ADD12C
	Matching rule 113	If PAON in PPD starts with number characters, which are not hyphenated, then flpaon3streetn5=ADDC10
	Matching rule 114	paonstreet1=ADD1C
	Matching rule 115	If PAON in PPD starts with number characters followed by an uppercase letter and ADD1 in EPC contains string pattern of ‘FLAT’ string followed by an uppercase letter, then paonstreet2=ADD5
	Matching rule 116	paonstreet2=apADD1
	Matching rule 117	paonstreet2=ADD1C2
	Matching rule 118	paonapstreet2=ADD13C2
	Matching rule 119	paonstreet3=ADDr66
Stage 4	Matching rule 120	saonpaonstreet2=ADDRE
	Matching rule 121	saonpaonstreet2=ADD12
	Matching rule 122	saonpaonstreetn=ADDC
	Matching rule 123	saonpaon65street=ADD12C
	Matching rule 124	saonpaon62cstreetn2=ADD13C
	Matching rule 125	saonpaonstreetn=ADD6
	Matching rule 126	saonpaonstreetn=ADDCC
	Matching rule 127	saonpaon61xstreet=ADD12C2
	Matching rule 128	saonpaon61xstreet=ADDREC
	Matching rule 129	saonpaon62cstreetn=ADD7
	Matching rule 130	saonpaonstreet1=ADD13C2
	Matching rule 131	If PAON does not start with number characters, saonpaon1=ADD1C9
	Matching rule 132	saonpaonn=ADDC4
	Matching rule 133	paonstreetn=ADDC4, then remove the incorrect matching for flats/maisonettes.
	Matching rule 134	For flats/maisonettes, saon2paon61street=ADDCC
	Matching rule 135	saonpaonn=ADD12C
	Matching rule 136	For flats/maisonettes, flsaonpaonstreet0=ADD
	Matching rule 137	For flats/maisonettes, flsaon1paonstreetn2=ADDCC
	Matching rule 138	For flats/maisonettes, flsaonpaonstreet1=ADDREC
	Matching rule 139	For flats/maisonettes, flsaonpaon62street1=ADDREC
	Matching rule 140	For flats/maisonettes, fldsaonpaonstreet1=ADDREC
	Matching rule 141	For flats/maisonettes, saon7paonstreet1=ADDRE
	Matching rule 142	For flats/maisonettes, saon7paonstreet2=ADDREC
	Matching rule 143	For flats/maisonettes, apsaonpaonstreet1=ADDREC
	Matching rule 144	For flats/maisonettes, saon7paonstreet2=ADD12C2
	Matching rule 145	For flats/maisonettes, apsaonpaonstreet1=ADD12C2
	Matching rule 146	saon7paonstreetn=ADDC4
	Matching rule 147	saon7paonn=ADD12C4
	Matching rule 148	saon4paonstreetn=ADDC4
	Matching rule 149	apsaonpaon6streetn=ADDC4
	Matching rule 150	For flats/maisonettes, flsaonpaonstreetn=ADDC4
	Matching rule 151	If PAON in PPD data does not start with number characters, saon4paonstreetn3=ADDC5
	Matching rule 152	saon4paonstreetn4=ADD12C
	Matching rule 153	For flats/maisonettes, saon4paonstreetn1=ADD12C
	Matching rule 154	For flats/maisonettes, if SAON contains the ‘APARTMENT’ string, then saon1paonstreetn=ADDC
	Matching rule 155	For flats/maisonettes, if SAON contains the ‘APARTMENT’ string, then saon1paonstreetn=ADD12C
	Matching rule 156	For flats/maisonettes, if SAON contains the ‘APARTMENT’ string, then saon1paonstreetn1=ADDC
	Matching rule 157	For flats/maisonettes, if SAON contains the ‘APARTMENT’ string, then saon1paonstreetn1=ADD12C
	Matching rule 158	For flats/maisonettes, if SAON contains the ‘APARTMENT’ string, then saon1paonstreetn2=ADDC3
	Matching rule 159	For flats/maisonettes, if SAON contains the ‘APARTMENT’ string, then saon1paonstreetn2=ADD12C1
	Matching rule 160	For flats/maisonettes, if SAON contains the ‘APARTMENT’ string, then saon2paon61street=ADD12C
	Matching rule 161	For flats/maisonettes, if SAON contains the ‘APARTMENT’ string, then saon2paonstreetn3=ADDC
	Matching rule 162	For flats/maisonettes, if SAON contains the ‘APARTMENT’ string, then saon2paonstreetn3=ADD12C
	Matching rule 163	For flats/maisonettes, if SAON containing the ‘APARTMENT’ string and paon does not start with numbers in PPD, saon2paonstreetn2=ADDC
	Matching rule 164	For flats/maisonettes with SAON contains the ‘APARTMENT’ string and PAON does not start with number characters, then saon2paonstreetn2=ADD12C
	Matching rule 165	For flats/maisonettes, if SAON contains the ‘APARTMENT’ string, then saonn2paonn1=ADDC
	Matching rule 166	If SAON contains the ‘APARTMENT’ string, then saonpaon62street=ADD12C
	Matching rule 167	If SAON contains the ‘APARTMENT’ string, then saon1paonstreet6n1=ADD12C
	Matching rule 168	If SAON contains the ‘APARTMENT’ string, then saon2paonstreetn=ADD12C
	Matching rule 169	If SAON contains the ‘APARTMENT’ string, then saonn3paonnstreet=ADD13C
	Matching rule 170	If SAON contains the ‘APARTMENT’ string, then saonn2paonn1streetn=ADDC
	Matching rule 171	For flats/maisonettes, if SAON contains the ‘APARTMENT’ string, then paon62saonpstreet=ADDREC
	Matching rule 172	If SAON contains the ‘APARTMENT’ string, then saonpaon62streetn1=ADDC
	Matching rule 173	If SAON contains the ‘APARTMENT’ string, then saon1paonstreet6n=ADD12C
	Matching rule 174	For flats/maisonettes, if SAON contains the ‘APARTMENT’ string, then saon2paonstreetn4=ADDC
	Matching rule 175	For flats/maisonettes, if SAON contains the ‘APARTMENT’ string, then saon2paonstreetn4=ADD12C
	Matching rule 176	For flats/maisonettes, if SAON contains the ‘APARTMENT’ string, then saon2paonstreetn4=ADD1num2
	Matching rule 177	For flats/maisonettes, saon5paonstreetn1=ADDC
	Matching rule 178	For flats/maisonettes, if SAON contains the pattern of ‘APARTMENT’ string followed a uppercase letter, then paonsaon2streetn=ADD1C
	Matching rule 179	For flats/maisonettes, if SAON contains the ‘APARTMENT’ string, then saon2paonstreetn2=ADD13C
	Matching rule 180	If SAON contains the ‘APARTMENT’ string, then saonpaon66street=ADDC6
	Matching rule 181	For flats/maisonettes, if SAON contains the ‘APARTMENT’ string, then saon1paonstreetn3=ADD12C
	Matching rule 182	For flats/maisonettes, if SAON contains the ‘APARTMENT’ string, then saon2street=ADDC
	Matching rule 183	If SAON contains the ‘APARTMENT’ string in PPD, then saon1paonstreet=ADDRE
	Matching rule 184	For flats/maisonettes, if SAON contains the ‘APARTMENT’ string, then saon2paonlo=ADDRE
	Matching rule 185	If SAON contains the ‘APARTMENT’ string, then saon1paon=ADD12
	Matching rule 186	For flats/maisonettes, if SAON contains the ‘APARTMENT’ string, then saon1paon61street=ADD12
	Matching rule 187	For detached, semi-detached and terrace houses, if SAON contains the ‘APARTMENT’ string, then saon2paonstreet=ADD12
	Matching rule 188	For flats/maisonettes, if SAON contains the ‘APARTMENT’ string, then saon1paon1=ADD1C9
	Matching rule 189	For flats/maisonettes, if SAON contains the ‘APARTMENT’ string, then saon1paonstreetn2=ADD12C2
	Matching rule 190	For flats/maisonettes, if SAON contains the ‘APARTMENT’ string, then psaonpaonstreet=ADDREC
	Matching rule 191	For flats/maisonettes, if SAON contains the ‘APARTMENT’ string, then saon2paon62street=ADD12
	Matching rule 192	If SAON in PPD contains the ‘APARTMENT’ string and the ADD2 in EPC contains a string pattern of hyphenated numbers, then saon2paonstreet=ADD1262
	Matching rule 193	saonpaonstreetn2=ADD7
	Matching rule 194	For flats/maisonettes, if PAON does not contains commas, then flsaonpaonstreet=add1f61f2
	Matching rule 195	If PAON starts with number characters and SAON ends with the string pattern of ‘FLAT’ string followed by an uppercase letter, then psaon8street=ADDREC
	Matching rule 196	saonpaonstreet1=add12643
	Matching rule 197	For detached, semi-detached and terrace houses, saonstreet=ADDRE
	Matching rule 198	saonstreetlo=ADDRE
	Matching rule 199	If SAON starts with number characters, unsaonpaonstreet2=ADDRE
	Matching rule 200	For flats/maisonettes, flsaonpaonstreet2=ADD8
	Matching rule 201	For flats/maisonettes, flsaonpaon1=ADD1C9
	Matching rule 202	For flats/maisonettes, saonpaon1=fladd
	Matching rule 203	For flats/maisonettes, saonpaon1=fladd1c
	Matching rule 204	For flats/maisonettes, if SAON contains 'FLAT' string, then saonpaonstreet3=fladd
	Matching rule 205	For flats/maisonettes, saon7paon6street=ADDRE
	Matching rule 206	For flats/maisonettes, saon7paon6street=ADD12
	Matching rule 207	saon8paonstreet2=ADDRE
	Matching rule 208	For flats/maisonettes, if PAON does not start with numbers characters, then saonpaonstreet2=fladd
	Matching rule 209	For street in PPD is null, paonlo=ADD12
	Matching rule 210	For flats/maisonettes, saonpaonstreet1=adddap
	Matching rule 211	For flats/maisonettes, if PAON does not start with numbers characters, then saonpaon61xstreet=fladdc
	Matching rule 212	For flats/maisonettes, saonpaon2=fladdc
	Matching rule 213	For flats/maisonettes, if SAON contains the ‘APARTMENT’ string, then saonpaonstreet5=apadd1632
	Matching rule 214	For flats/maisonettes, saonpaonstreet11=ADD12
	Matching rule 215	For flats/maisonettes, if PAON does not start with number characters, then saonpaon61xstreet=ADD1262C, then keep paon62 contains a string pattern of hyphenated numbers
	Matching rule 216	For flats/maisonettes, if PAON does not start with number characters, then saonpaon61street=ADD1262C, then keep add261 contains a string pattern of hyphenated numbers
	Matching rule 217	For flats/maisonettes, flsaonpaonstreet3=ADD12C5
	Matching rule 218	For flats/maisonettes, flsaonpaonstreet3=ADD12C1
	Matching rule 219	For flats/maisonettes, saonstreet1=ADD1C7
	Matching rule 220	For flats/maisonettes, if SAON contains a hyphen and PAON starts with number characters, then saonpaonstreet1=add1f61f3
	Matching rule 221	For flats/maisonettes, if SAON contains a hyphen and PAON starting with number characters and ADD in EPC data contains a hyphen, saonpaon62street=ADDRE
	Matching rule 222	For flats/maisonettes, saonstreet2=ADD1264
	Matching rule 223	For flats/maisonettes, flsaonpaon61street=ADDREC
	Matching rule 224	For flats/maisonettes, if SAON contains the ‘FLAT’ string, then saon4paonstreet=ADD12
	Matching rule 225	For flats/maisonettes, if SAON contains a hyphen, saonpaon61street1=ADD1263
	Matching rule 226	For flats/maisonettes, flsaonpaon2=ADDRE
	Matching rule 227	saonpaon3=ADD1
	Matching rule 228	For flats/maisonettes, saonstreet3=ADDC
	Matching rule 229	For flats/maisonettes, flsaonpaon3=ADD12
	Matching rule 230	For flats/maisonettes, flsaonpaonstreet4=ADD1263
	Matching rule 231	If flats/maisonettes, if PAON does not start with number characters but contains numbers and commas, then if saonstreet=ADD1265, keep the results for the postcode having the same add2.
	Matching rule 232	paonsaonstreet=ADDRE
	Matching rule 233	For flats/maisonettes, if PAON contains a hyphen, saonpaon61=ADD12
	Matching rule 234	For flats/maisonettes, saon7paon=ADD12
	Matching rule 235	For flats/maisonettes, paonsaonstreet1=ADD12
	Matching rule 236	For flats/maisonettes, flsaonpaon61street1=ADD12
	Matching rule 237	For flats/maisonettes, apsaonpaon=ADD12C6
	Matching rule 238	For flats/maisonettes, saon1paon62street=ADD12
	Matching rule 239	For flats/maisonettes, if SAON contains the ‘APARTMENT’ string and PAON does not start with number characters, saonstreet=ADDC5
	Matching rule 240	For flats/maisonettes, apsaonpaon62street1=ADDC8
	Matching rule 241	Having corrected the mismatched address strings in EPC or PPD, then saonpaonstreet2=ADDRE
	Matching rule 242	For flats/maisonettes, saon2paonstreet1=ADDC9
	Matching rule 243	For flats/maisonettes, apsaonpaonstreet2=ADD1262cc
	Matching rule 244	For flats/maisonettess, psaonpstreet=ADDRE
	Matching rule 245	saonpaon65street1=ADD12C
	Matching rule 246	For flats/maisonettes, saon2paonstreetn3=ADDC
	Matching rule 247	saonpaonn=ADD12C
	Matching rule 248	saon1paonstreetn1=ADDC
	Matching rule 249	For flats/maisonettes, if PAON does not start with number characters in PPD and ADD in EPC does not contain a hyphenated number string, saon4paonstreetn1=ADDC4
	Matching rule 250	For flats/maisonettes, saon1paonstreetn=ADDC4
	Matching rule 251	saonpaonlon=ADDC4

^1^In this column, variables on the left side of the symbol (=) refer to address fields in the LR-PPD, variables on the right side of the symbol (=) refer to address fields in the Domestic EPCs. Symbol (=) refers to string matching function.
